# Inhibitors of the Machupo Virus L Endonuclease for Bolivian Hemorrhagic Fever Treatments

**DOI:** 10.3390/microorganisms14061377

**Published:** 2026-06-22

**Authors:** Oluwafoyinsola O. Faniyi, Kristin V. Lyles, Neva Agarwala, Haozhe Cheng, Elise Copeland, Teri Tran, Shuyue Yang, Bingchen Yu, Binghe Wang, Xiaoxiao Yang, Ming Luo

**Affiliations:** 1 Department of Chemistry, Georgia State University, Atlanta, GA 30302, USA; ofaniyi1@gsu.edu (O.O.F.); kvanmouwerik1@gsu.edu (K.V.L.); agarwalaneva@gmail.com (N.A.); ecopeland6@student.gsu.edu (E.C.); teritran1001@gmail.com (T.T.); syang40@student.gsu.edu (S.Y.); byu8@gsu.edu (B.Y.); bwang31@gsu.edu (B.W.); shawnyang.ga@gmail.com (X.Y.); 2 Center for Diagnostics and Therapeutics, Georgia State University, Atlanta, GA 30302, USA; 3 Department of Biology, Georgia State University, Atlanta, GA 30302, USA; hcheng11@student.gsu.edu

**Keywords:** machupo virus, arenaviridae, L endonuclease, antiviral, cap snatching

## Abstract

Machupo virus (MACV) is the causal agent of Bolivian Hemorrhagic fever. It is highly pathogenic, has a high mortality rate, and currently lacks specific treatments or vaccines. MACV belongs to the *Arenaviridae* family, which uses a cap-snatching mechanism during the transcription process. Its viral polymerase, the L protein, harbors the endonuclease activity required for cap snatching, making it a suitable target for the development of antiviral therapeutics. We combined experimental and computational methods to characterize MACV endonuclease activity and evaluate inhibitors. A fluorescence resonance energy transfer (FRET) assay was used to measure the enzymatic activity of endonuclease and identify potent inhibitors via high-throughput screening. FRET assays identified BW-148, an inhibitor with a 48.4 µM (95% CI: 37.3–59.3 µM; R^2^ = 0.98) IC_50_, and a K_D_ of 13.7 µM (95% CI: 8.2–19.2 µM, n = 3). Docking studies reveal that BW-148 may bind near the MACV endonuclease catalytic site, inhibiting enzymatic activities by metal chelating. BW-148 is a useful lead compound for further optimization of Machupo endonuclease inhibitors.

## 1. Introduction

Machupo virus (MACV) is a biosafety level 4-classified New World Arenavirus in the Order *Bunyavirales.* It was first isolated from the spleen of a patient suffering from Bolivian Haemorrhagic Fever (BHF) during the 1963–1964 outbreak in Bolivia and South America [1,2,3,4]. The primary carrier of MACV is the field mouse *Calomys callosus*, although mosquitoes, ticks, and humans can act as secondary carriers of MACV [5,6]. Infection usually occurs by accidentally inhaling tiny particles from the rodent’s urine, droppings, or saliva (aerosol transmission), by eating contaminated food, or by direct contact with these materials.

BHF illness typically starts 3–16 days after exposure, with flu-like symptoms, progressing to bleeding, tremors, seizures, and coma in severe cases [7]. BHF infections have mortality rates of up to 35%, and treatment is limited to supportive care [4,7]. Although the antiviral drug ribavirin has been used to treat infections at early stages, there is no strong evidence that it is effective for BHF, perhaps due to the rarity of the disease [7,8]. MACV is an enveloped virus encapsulating a bi-segmented RNA genome that encodes four proteins: the nucleoprotein (NP), glycoprotein precursor (GPC), matrix zinc-binding protein (Z), and the large RNA-dependent RNA polymerase (L) [9,10,11]. The L protein is a single polypeptide chain of over 2000 amino acids that plays the key role in transcription and replication of the viral genome. Near atomic resolution Cryo-EM structures show that the L protein of MACV and Lassa virus (LASV), another arenavirus, adopt similar architecture, resembling the heterotrimeric influenza polymerase, with the N-terminal PA-like region, central PB1-like RdRp, and a C-terminal PB2-like region fused into a single polypeptide [12]. The N-terminal PA-like region of both MACV and LASV corresponds to the endonuclease (EN), responsible for cap snatching during the early stage of transcription [13,14]. Cap snatching involves the cleavage of host mRNA downstream of the cap to generate primers for viral mRNA synthesis. It is a conserved mechanism among segmented negative RNA viruses (SNSVs), including orthomyxoviruses and bunyaviruses [13,15].

MACV endonuclease is predominantly α helical in organization and structurally homologous to the cap endonucleases of other arenaviruses. The domain was sufficiently ordered in cryo-EM density map to permit fitting of the previously solved LASVEN structure, confirming conservation of canonical nuclease architecture [12]. Conservation of secondary structural elements and high similarity in the binding region were confirmed in our preliminary multiple sequence alignment and structural comparison between the EN of MACV and those of other arenaviruses, such as LASV, Junin virus (JUNV), and Lymphocytic choriomeningitis virus (LCMV). Previous studies also reveal structural conservation in the active site of SNSV endonucleases, including arenaviruses, which contain a PD(D/E)xK motif that coordinates divalent metal ions [13]. However, in arenaviruses, the histidine required for metal coordination is replaced by a glutamate (E51) [13]. This well-defined structural motif, its key relevance in transcription, and the enzymatic nature of the Machupo virus L endonuclease make it a prime antiviral target for developing BHF treatments.

Studies have reported that Baloxavir Marboxyl (BMX), a clinically prescribed drug for influenza virus infections, inhibits influenza EN activity [16,17,18,19,20]. Other groups have attempted to develop SNSV inhibitors using the BXM scaffold as a starting point [21,22,23,24]. Their results suggest that the derivatives of BMX are up to 1000-fold more potent than ribavirin against JUNV and LCMV to inhibit in vitro infection, with subnanomolar EC_90_, and one of the compounds reduced LASV replication by up to 3 log_10_ at 1 µM. Using a mouse model, the lead compound improved survival in LCMV-infected animals to 80–100% compared with 20% survival with ribavirin [21,22]. There are no reports on inhibitor studies on the Machupo virus L endonuclease (MACVEN). To identify inhibitors targeting the endonuclease of MACV L-polymerase, we purified the recombinant endonuclease domain of the L protein and performed FRET-based compound screening assays. We identified BW-148 as a lead of MACVEN inhibitors. Its binding to MACVEN was validated by a Monolith X spectral shift assay, and a potential binding site was observed by docking studies.

## 2. Materials and Methods

### 2.1. Protein Expression and Purification

The expression vector pET-28a-MACEN was purchased from GenScript USA Inc. (Piscataway, NJ, USA). The MACV endonuclease domain consists of the first 173 residues of the L protein (Uniprot ID: Q6IUF8). The sequence was optimized for expression in *Escherichia coli,* cloned into a pET-28a (+) vector with an added TEV cleavage site upstream of the N-terminal Histag. The vector was amplified in BL21Star^TM^ (DE3) *E.coli* strain (Invitrogen, Carlsbad, CA, USA). For expression, cells were grown in Luria broth with 70 µg/mL kanamycin at 37 °C with 225 rpm. When the culture reached an OD_600nm_ of 0.6–0.8, expression was induced with 0.2 mM Isopropyl β-D-1-thiogalactopyranoside (IPTG).

The culture was allowed to grow for 18 h at the reduced temperature of 16 °C with 180 rpm. Cells were harvested by centrifugation, and the cell pellet was stored at −80 °C. Cells were resuspended in lysis buffer containing 20 mM Tris-HCl, 150 mM NaCl, 10 mM Imidazole, 5 mM β-mercaptoethanol, at pH 8.0. The resuspension was stirred on ice in 1:10 *w*/*v* ratio of cells until the pellet fully thawed, and then treated with 1 mM phenylmethylsulfonyl fluoride (PMSF). Cells were lysed by sonication on ice (20% amplitude, 50 s on/10 s off, 15 min total). The solution was centrifuged at 20,000 rpm for 40 min at 4 °C, and then the lysate was further clarified using a 0.22 µM syringe filter. The clarified lysate was purified with a 1 mL HisTrap HP (Cytiva^TM^, Marlborough, MA, USA) column, and stored in buffer containing 20 mM Tris-HCl, 150 mM NaCl, pH 8.0, with 8% glycerol at −80 ° C.

### 2.2. FRET Endonuclease Activity Assay

EN activity assay was conducted by modifying a previously published protocol [25]. RNA analog sequence was AUUUUGUUUUUAAUAUUUC with a 5′ fluorescein amidite (FAM) and a 3′ black hole quencher 1 (BHQ1) made by Genscript USA Inc. (Piscataway, NJ, USA).

The reaction mixture was made up of 5 µM MACVEN in 50 µL assay buffer (20 mM Tris-HCl, 150 mM NaCl, 1 mM TCEP, pH 8.0) incubated with 2 mM MnCl_2_. We used 2 mM MnCl_2_, based on prior studies of a similar endonuclease, which showed that this concentration supported catalytic activity under comparable assay conditions [26]. To initiate EN activity, the reaction mixture was first transferred to the plate reader, then 0.5 µM RNA analog was added. The experiment was immediately run in a SpectraMax ID5 plate reader (Molecular Devices, San Jose, CA, USA) at 37 °C for 70 min. Every 6 min, the fluorescence was measured with excitation of 485 nm and emission of 535 nm. Afterwards, 10 µL of the sample was added to 10 µL loading buffer (formamide with 10 mM EDTA), which also halted the reaction. RNA cleavage was confirmed visually by running the samples on 20% polyacrylamide gels with 8M Urea (PAGE Urea gel) in 1× Tris Borate with EDTA (TBE) buffer for 60 min at 80 volts. Gels were imaged on a Safe Imager^TM^ 2.0 Blue Light Transilluminator (Invitrogen, Carlsbad, CA, USA). Graphs displaying increase in fluorescence intensity vs. time were prepared using Microsoft Excel, with increased fluorescence intensity being proportional to increasing enzymatic activity.

### 2.3. High-Throughput FRET EN Inhibitor Assay

Screening assays were set up similarly as 2.2: compounds were incubated with 5 µM MACV EN in assay buffer containing 2 mM MnCl_2_ and 0.5 µM RNA analog. Initial compounds were identified through in silico compound screening using Lassa virus endonuclease (LASVEN; PDB 5J1P) [13], an EN of similar structural homology to MACVEN. A total of 44 compounds were initially screened in vitro using MACVEN. Compound effect was evaluated by assessing any reductions in FRET intensity as a result of compound-EN incubation. As with the EN assay, the same plate reading system and parameters were used, and the samples were run on PAGE Urea gels and viewed with the Invitrogen Safe Imager 2.0 Blue Light Transilluminator. Graphs displaying results were prepared with Microsoft Excel.

### 2.4. Spectral Shift Measurement of Binding Affinity

The compound binding affinity of the endonuclease was determined by NanoTemper Technologies Monolith X by the Georgia State University Department of Chemistry, Mass Spectrometry Facility. Briefly, the MACVEN was labeled with NHS Red 2nd Generation dye (NanoTemper Technologies GmbH, Munich, Germany). Next, 50 nM of labeled MACVEN in assay buffer (10 mM Tris, 1 mM MnCl_2_, 150 mM NaCl, 1 mM TCEP, 2% n-Octyl-β-D-glycopyranoside, pH 8.0, and 10% DMSO) was incubated at room temperature for 30 min, with a concentration range of BW-148 between 300 µM and 9 nM to determine the K_D_. The samples were loaded into capillaries, and fluorescence intensity and binding affinity (K_D_) were measured using a Monolith X (NanoTemper Technologies) and run in triplicate. The experiment was also repeated without MnCl_2_ in the assay buffer.

To evaluate the enzyme’s substrate interaction, an initial assay was run using 8 nM fluorescent-labeled CY5 RNA (5′-CY5-AUUUUGUUUUUAAUAUUUC-3) in assay buffer, replacing the metal with 10 mM EDTA to avoid RNA cleavage. The EC_50_ value was determined from the spectral shift output. A maximum concentration of 30 µM MACVEN was used for this assay. Raw fluorescence emission was recorded at 650 nm and 670 nm as a function of the binding partners’ concentrations [27].

### 2.5. Docking Studies for Inhibitor–Protein Complex

MACVEN structure was generated from its 3.58 Å Cyro-EM full-length L protein structure (PDB 6KLD) [12] with residues 1–173 (Uniprot ID: Q6IUF8) using SWISS-MODEL [28].

Docking experiments were also performed with both MACVEN and LASVEN (PDB 5J1P) structures. AutoDock 4.2 and AutoDock Tools (ADT) were used to prepare the protein and ligand files [29]. Polar hydrogen atoms and Kollman partial charges were assigned before saving macromolecules in PDBQT format. Ligand structures were drawn in ChemDraw Professional 25.5, converted to three-dimensional structures, and geometrically optimized using Avogadro 1.2. The ligand was further optimized using the Autodock4 force field. Gasteiger partial charges and polar hydrogens were also added, and all rotatable bonds were defined.

The grid box dimensions were set to 40 × 50 × 48 for MACVEN and 48 × 40 × 40 for LASVEN, with a grid spacing of 0.375 for both proteins. The grid center was positioned based on known arenavirus active-site residues, polar hydrogens were added to the simulated proteins, and Kollman united-atom partial charges were assigned to the protein structures.

## 3. Results

### 3.1. Endonuclease Activity

**Figure 1:**
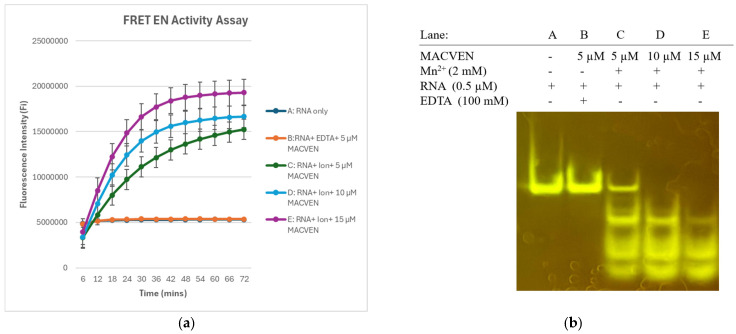
MACVEN activity assay: (**a**) time-dependent RNA cleavage measured by FRET with increasing concentrations of MACVEN; (**b**) reaction products analyzed in 20% denaturing polyacrylamide/ 8 M Urea PAGE gels and visualized by fluorescence imaging.

FRET EN assay confirms MACVEN activity at varied concentrations of 5–15 µM (Figure 1a,b). The initial reaction was rapid, with fluorescence doubling in the first few minutes before slowing. Looking at the RNA products on the PAGE Urea gel, we see that at 10 and 15 µM, the top band with the full-length RNA substrate is completely cleaved into three smaller bands (Figure 1b). When the endonuclease concentration was reduced to 5 µM, uncleaved RNA remained. We decided to use 5 µM for our inhibitor screen since it provided robust fluorescence readings and would require less of our compounds than at higher endonuclease concentrations.

### 3.2. BW-148 Inhibits Machupo Virus Endonuclease Activity

**Figure 2:**
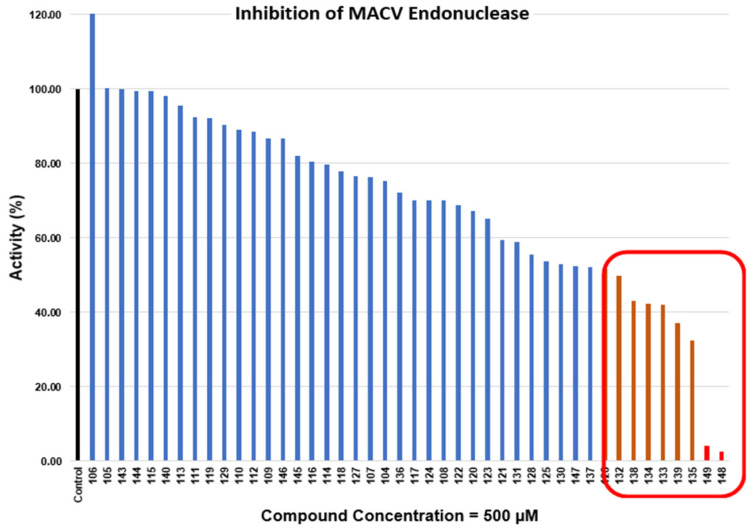
Comparison of the effects of various compounds on the activity of Machupo virus endonuclease (compounds highlighted in red were the most effective inhibitors at 500 µM concentration).

A preliminary screen was conducted by testing a total of 44 compounds with purified MACVEN (Figure 2, Table 1, and Supplemental Table S1). BXA was not included as a control in our assay because previous studies reported no antiviral activity against arenaviruses [21]. We incubated our compounds with 5 µM of MACVEN and its cofactor, before adding the RNA. Real-time FRET EN assays were used to examine the reduction in EN activity for increasing durations of incubation. Using the FRET data, we calculated % inhibition by the compound compared to the non-treated control using the equation:

$$\%\;\mathrm{inhibition}\;=\left(1-\left(\frac{\Delta Fi}{\Delta Ft}\right)\right)\times 100.$$

where ΔFi is net increase in fluorescence intensity with inhibitors, and ΔFt is net change in fluorescence intensity without inhibitors.

**Figure 3:**
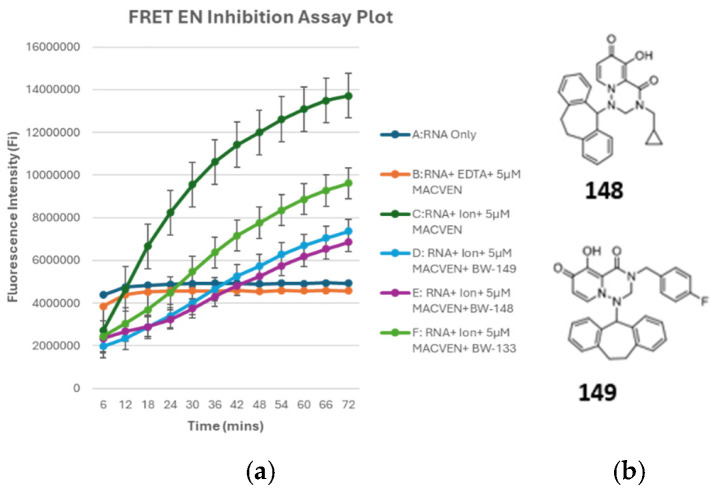
FRET endonuclease inhibition analysis: (**a**) time-dependent RNA cleavage measuring the reduction in MACVEN FRET intensity when incubated with 100 µM of BW-148, BW-149, and 500 µM BW-133; (**b**) chemical structure of BW-148 (**top**) and BW-149 (**bottom**).

**Figure 4:**
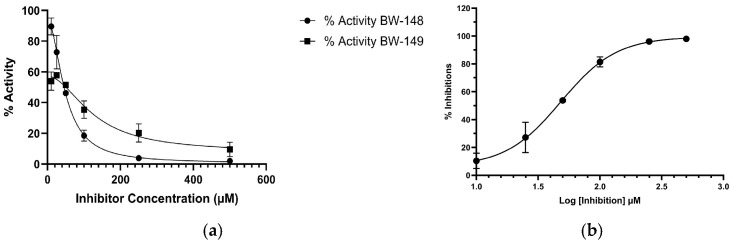
Concentration dependent inhibition of MACVEN: (**a**) dose-dependent response curve for BW-148 and BW-149; (**b**) IC_50_ plot for compound 148.

We identified eight compounds that reduced EN activity by greater than 50% and termed them effective at inhibiting the MACVEN (Table 1). The remaining 36 compounds were deemed non-inhibitory (Supplemental Table S1). By this screen, we identified two compounds, BW-148 and BW-149, that showed greater inhibitory effects than all other compounds (Figure 2 and Figure 3, Table 1). BW-148 produced 97% inhibitions at 500 µM, while BW-149 produced 96% inhibitions at the same concentration (Table 1, Figure 4a,b). To determine the half-maximal inhibitory concentration (IC_50_), we tested both compounds using 10, 25, 50, 100, 250, and 500 µM to generate a dose-dependent curve with GraphPad Prism 11. We identified BW-148 as the most effective inhibitor, with an IC_50_ of 48.4 µM (95% CI: 37.3–59.3 µM; R^2^ = 0.98) (Figure 4b). The IC_50_ for BW-149 was 154.8 µM (95% CI: 143.3–166.8; R^2^ = 0.99) (Supplemental Figure S1).

### 3.3. Spectral Shift Analysis of BW-148 Bound to MACVEN

**Figure 5:**
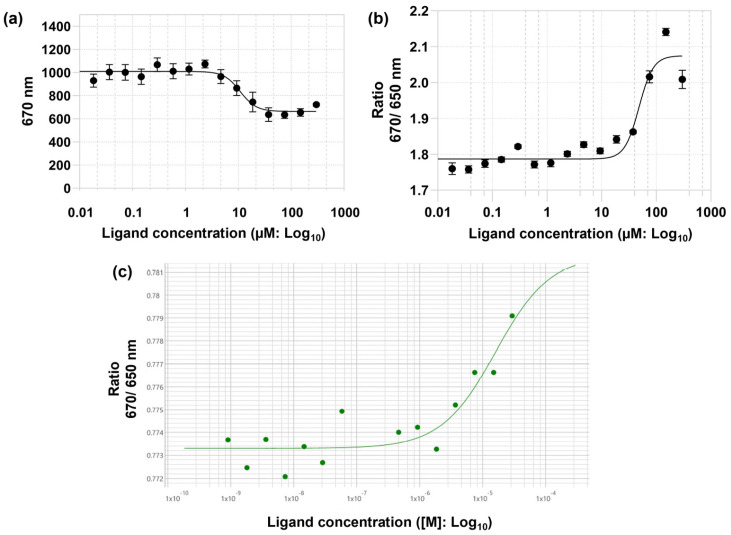
MACVEN time-dependent maturation with spectral shift: (**a**) following a 30 min incubation, binding of NHS-labeled MACVEN with BW-148 shows K_D_ = 13.7 µM in the presence of 1 mM MnCl_2_; (**b**) following a 30 min incubation, binding of NHS-labeled MACVEN with BW-148 shows K_D_ = 16.3 µM without MnCl_2_; (**c**) following a 30 min incubation, interaction between MACVEN and CY5-RNA shows EC_50_ = 16.0 µM for MACVEN binding to RNA.

BW-148 was further investigated to determine its binding affinity for MACVEN labeled with the fluorophore NHS Red. Binding of BW-148 to MACVEN was monitored via raw fluorescence at 670 nm, resulting in a K_D_ of 13.7 µM (CI: 8.2–19.2 µM, n = 3) in the presence of 1 mM MnCl_2_ (Figure 5a). However, the characteristic 670/650 nm spectral shift curve was absent, possibly due to metal interference with the fluorophore’s emission profile. In the absence of MnCl_2_, the 670/650 nm spectral shift was present and indicated a K_D_ of 16.3 µM (CI: 9.3–23.3 µM, n = 3) (Figure 5b).

The spectral shift data suggests that BW-148 binds MACVEN tightly (Table 2). An initial experiment was also run to establish a baseline for MACVEN protein interaction with a CY5-labeled RNA (8 nM). Up to 30 µM of MACVEN was used in this assay with 30 min pre-incubation. A rightward shift with an EC_50_ of 16 µM MACVEN (S/N = 11) was observed for MACVEN binding to RNA (Figure 5c). This experiment showed that MACVEN recognizes its substrate modestly.

### 3.4. Interaction of BW-148 with the L Endonuclease Protein of Arenaviruses

**Figure 6:**
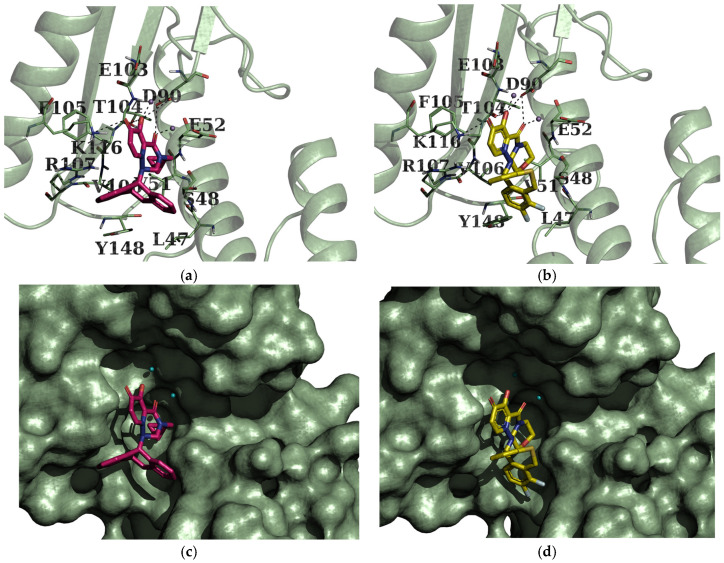
Binding mode and molecular interactions of compounds BW-148 and BXA to MACVEN. (**a**) Three-dimensional representation of MACVEN-BW-148 interaction at surface level; (**b**) three-dimensional representation of MACVEN-BXA interaction at surface level; (**c**) surface representation of MACVEN-BW-148 interaction within the active site; (**d**) surface representation of MACVEN-BXA docked with MACVEN.

Docking studies suggest that BW-148 interacts with MACVEN by binding to its active-site cleft with a predicted binding energy of −6.55 kcal/mol. The studies also indicate that BW-148 may interact with two Mn^2+^ ions, form polar contacts with residues D90 and K116, and form main-chain interactions with T104. BW-148 is also predicted to form hydrophobic interactions with surrounding residues, along with strong cation-π stacking between the guanidinium groups of R107 and K116 and the thiepane moiety of the compound (Figure 6a). The presence of a cyclopropylmethyl group in the triazinone core also enables nonpolar interactions with V51, E52, and V106 of the enzyme. Similar interactions were seen when comparing interactions with docked Baloxavir acid (BXA), the active ingredient of Baloxavir Marboxyl, except for polar interactions with S48 (Figure 6b). Our docking studies with LASVEN (PDB 5J1P) suggest that BW-148 yields a binding free energy of −7.59 kcal/mol. The stabilizing interactions of the complex were the coordination with one of the two Mn^2^+ ions, polar interactions with residues D89, K115, K112, E102, and D66, and main-chain interactions with V87 and P88 (Supplemental Figure S2).

Since the endonuclease is part of the full-length L protein responsible for transcription of the arenavirus genome, we conducted superimposition studies using Cryo-EM full-length L protein (PDB 6KLD) [12] to explore potential inhibitory effects and mechanistic implications within the full-length protein (Figure 6a–c).

**Figure 7:**
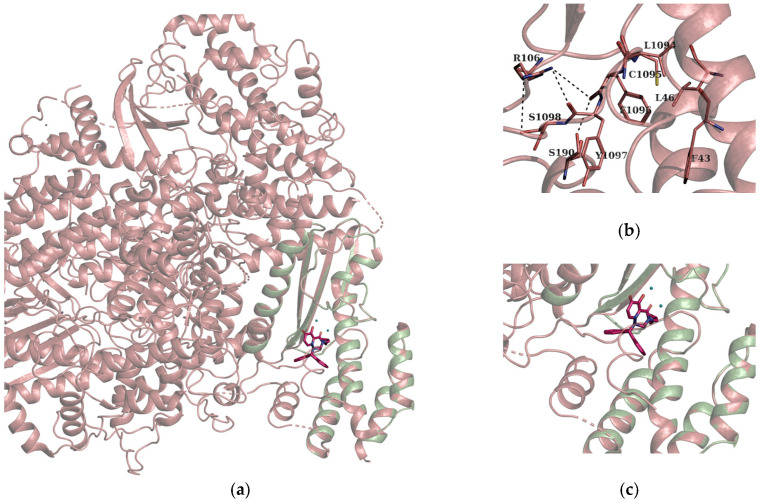
Superimposition studies of MACVEN with full-length L protein. (**a**) Superposition of BW-148-MACVEN complex (light green) with the apo form of Machupo L protein (pink PBD 6KLD); (**b**) interaction between endonuclease domain residues (F44, L46, R106, S190) and PB1-like domain residues (LCFYS motif 1094–1098); (**c**) close up of MACV L protein (wheat) loop closing the active-site region where BW-148 (pink) is bound to MACVEN (light green).

Superimposition studies (Figure 7a–c) suggest that BW-148 may bind within the same region where an inhibitory loop, i.e., where the 1096-LCFYS motif binds to the endonuclease active-site pocket, could block substrate access at a pre-initiation stage; this may be the case when the endonuclease is active, and the loop is open. This conclusion is based on the architectural conservation of Lassa and Machupo L proteins [12], as detailed structural studies of the Lassa L protein in its apo, promoter-bound, and RNA synthesis states revealed that the endonuclease switches between inhibited and uninhibited states due to associated conformational changes in the L protein [30]. In the apo state, endonuclease is blocked by residues 1092–1105 of the PB1-like region, where the arenavirus conserved 1096-LCFYS motif binds to the endonuclease active-site pocket, preventing substrate RNA from binding [30]. Further experimental validation can be used to confirm these mechanisms.

## 4. Discussion

We have investigated compounds to identify potential inhibitors of MACVEN. From a screen of 44 compounds, we identified 8 compounds that showed inhibitory effects against the endonuclease. Two of these compounds moderately inhibited the endonuclease, as confirmed by our FRET assays. We used the spectral shift assay to elucidate the binding mechanism of our lead inhibitor, BW-148. BW-148 shows a K_D_ of 13.7 µM (95% CI: 8.2–19.2 µM) and an IC_50_ of 48.4 µM (95% CI: 37.3–59.3 µM; R^2^ = 0.98) in FRET assays.

Docking studies revealed a potential binding mode of BW-148 to both MACVEN and LASVEN. The studies suggest that BW-148 may form coordination with two Mn^2^+ metal ions in MACVEN, polar contacts with residues D90, K116, and main-chain atoms of T104. It also forms hydrophobic interactions with surrounding residues, and cation-π stacking by the guanidinium group of R107 and the amino group of K116 with thiepane moiety of the compound. The presence of the cyclopropylmethyl group in the triazinone core enables hydrophobic interactions with the protein. The binding interaction indicates active-site occupation by BW-148, which likely favors competitive inhibition, a mechanism similar to that of BXA inhibition.

BXA is a first-in-kind endonuclease inhibitor that treats influenza virus infections [16,17,18,24,31]. The rigid structure of BXA (Supplemental Figure S3) helps it fit precisely into the endonuclease’s active-site pocket. One of the most important structure features is the β-diketone-like moiety of which oxygen atoms chelate the catalytic metal ions (usually Mn^2+^) present in the endonuclease active site [17,19,32,33]. The -OH group participates in hydrogen bonding with residues at the active site. It also participates in the metal chelation. Phenyl-type rings provide π–π stacking and hydrophobic interactions with residues in the enzyme pocket. By chelating the divalent metal active site, BXA binds in the active site of EN to block cleavage of the host mRNA [17,34]. Our compound, BW-148, is a derivative of BXA with a cyclopropylmethyl group at position 7, without S and F atoms in the thiepane core.

To explore plausible inhibitory action and mechanistic implications of BW-148 against MACVEN, we studied the full-length L protein and superimposed our EN Inhibitor complex with the full-length L protein (Figure 7a–c) using the Cryo-EM structure of the Machupo virus L protein, PDB 6KLD [12].

Structural studies of Lassa L protein report that in its apo, promoter-bound, and RNA synthesis states, the endonuclease switches between inhibited and uninhibited states due to conformational changes of the L protein [30]. In the apo state, the endonuclease active site is blocked by residues 1092–1105 of the PB1-like region, where the arenavirus conserved 1096-LCFYS motif binds to the endonuclease active-site pocket, preventing substrate RNA binding [30]. A similar interaction may be observed in the MACV-apo L protein due to conserved architectural similarity [12]. In the distal promoter-bound pre-initiation state, the endonuclease undergoes an approximately 160 ° flip to the solvent-exposed state, free of the inhibitory peptide loop region [30]. Again, in the elongation state, the endonuclease is stabilized in a third position where the active site is inhibited by its own helix (181–188), interacting with E51, D89, and two cations [30]. From the strong interactions established by BW-148, we propose that due to conservation of the RdRp architecture of Lassa L protein and Machupo L protein, BW-148 could bind an uninhibited, solvent-exposed state of the endonuclease in the pre-initiation state. If it is required that a MACVEN inhibitor competes with the 1096-LCFYS motif in the apo state of the L protein, the conformation of the inhibitory loop needs to be changed, which is possible because the loop is connected to a flexible region (structurally disordered) of the L protein [30]. BXA derivatives have been widely explored by researchers for developing EN inhibitors against *Bunyavirales*, including arenaviruses (LASV, LCMV, and JUNV). Modifications to the triazinanone core at the 3rd and 7th positions of BXA, and addition of the cyclopropylmethyl group at the 7th position and carbonyl group at position 3 by previous researchers showed the possibility of reducing infections in LCMV, LASV and Lacrosse virus (LACV) in vivo [21,22,23]. Our docking studies thoroughly examined the binding mode of BXA derivatives. Identification of Tashionine I and its analog, Tashionine II, as cap-dependent endonuclease inhibitors with broad-spectrum antiviral activity against viruses in the order *Bunyavirales* has been previously reported [35].

## 5. Conclusions

In conclusion, this study suggests that BW-148 likely exerts competitive inhibition on arenavirus endonucleases by binding at the active site, blocking substrate RNA binding. This is similar to previously reported inhibitors, such as BXA. As a BXA derivative, BW-148 chelates divalent metals (Mn^2+^/Mg^2+^) in the active site and is involved in extensive polar and hydrophobic interactions with active-site residues. BW-148 is a useful lead compound for the future development of potent MACVEN inhibitors.

## Figures and Tables

**Table 1:** Comparison of compounds showing effective inhibition at 500 µM concentrations.

Compound ID	Structure	ΔFt	ΔFi	Inhibition % $\left(1-\left(\frac{\Delta Fi}{\Delta Ft}\right)\right)\times 100$	Gel Images
Control		1,242,943		-	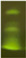
BW-132	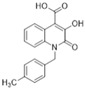	1,109,981	553,294	50.15%	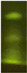
BW-133	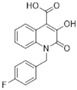	1,242,943	522,710	57.95%	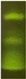
BW-134	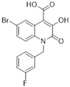	1,109,981	468,673	57.78%	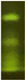
BW-135	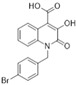	1,109,981	359,661	67.60%	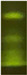
BW-138	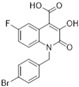	1,109,981	477,629	56.97%	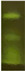
BW-139	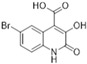	1,109,981	411,539	62.92%	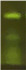
BW-148	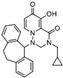	1,109,981	29,678	97.41%	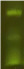
BW-149	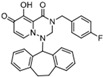	1,144,674	45,712	95.88%	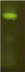

**Table 2:** Comparative analysis of inhibitor binding affinity (K_D_) and RNA functional efficiency EC_50_.

Interaction Pair	Experimental Setup	EC_50_ or K_D_	S/N
CY5-RNA + MACVEN	8 nm RNA	16.0 µM	10.9
	30 µM MACVEN		
	(30 min incubation)		
BW-148 + MACVEN	50 nM MACVEN	13.7 µM	>5.5
	300 µM BW-148		
	30 min incubation		
BW-148 + MACVEN	50 nM MACVEN	16.3 µM	>7.7
	300 µM BW-148		
	30 min incubation		

## Data Availability

The original contributions presented in this study are included in the article/Supplementary Material. Further inquiries can be directed to the corresponding author.

## Supplementary Materials

The following supporting information can be downloaded at: https://www.mdpi.com/article/10.3390/microorganisms14061377/s1. Table S1. Compounds showing no effective inhibition at 500 µM concentrations. Figure S1: IC50 of BW-149. Figure S2: Complexes of endonucleases with docked compounds. Active site interactions of LASVEN (light blue) with (a) BW-148 (orange) and (b) BXA (yellow) are shown. Figure S3: Structure of Baloxavir acid (BXA).

## Abbreviations

The following abbreviations are used in this manuscript:

BHF	Bolivian Hammorhagic Virus
BXA	Baloxavir Acid
BXM	Baloxavir Marboxyl
Cryo-Em	Cryo Electron Microscopy
DMSO	Dimethyl Sulfoxide
EDTA	Ethylenediaminetetraacetic Acid
*E. coli*	Escherichia Coli
EN	Endonuclease
FRET	Fluorescence Resonance Energy Transfer
GPC	Glycoprotein Precursor
MACV	Machupo Virus
MACVEN	Machupo Virus Endonuclease
NP	Nucleoprotein
OD600	Optical Density at 600 nm
LASV	Lassa Virus
LASVEN	Lassa Virus Endonuclease
LCMV	Lymphocytic Choriomeningitis Virus (LCMV)
PDB	Protein Data Bank
SNSVS	Segmented Negative Strand RNA Virus
