# Supplementary material for: Inhibitors of the Machupo Virus L Endonuclease for Bolivian Hemorrhagic Fever Treatments
Source: Microorganisms. 2026 Jun 22;14(6):1377. doi: 10.3390/microorganisms14061377 (PMC13305936; doi:10.3390/microorganisms14061377)
Supplement: Supplementary file 1 [file microorganisms-14-01377-s001.zip › microorganisms-4260969-supplementary.pdf]

Supporting Information for

Inhibitors of the Machupo Virus L Endonuclease for Bolivian Hemorrhagic Fever Treatments

Oluwafoyinsola O. Faniyi [1,2], Kristin V. Lyles [1], Neva Agarwala [1], Haozhe Cheng [3], Elise Copeland [1], Teri Tran [1], Shuyue Yang [1], Bingchen Yu [1,2], Binghe Wang [1,2], Xiaoxiao Yang [1] and Ming Luo [1,2,*]

[1] Department of Chemistry, Georgia State University, Atlanta, GA 30302, USA; ofaniyi1@gsu.edu (O.O.F.); kvanmouwerik1@gsu.edu (K.V.L.); agarwalaneva@gmail.com (N.A.); ecopeland6@student.gsu.edu (E.C.); teritran1001@gmail.com (T.T.); syang40@student.gsu.edu (S.Y.); byu8@gsu.edu (B.Y.); bwang31@gsu.edu (B.W.); shawnyang.ga@gmail.com (X.Y.)
[2] Center for Diagnostics and Therapeutics, Georgia State University, Atlanta, GA 30302, USA
[3] Department of Biology, Georgia State University, Atlanta, GA 30302, USA; hcheng11@student.gsu.edu
* Correspondence: mluo@gsu.edu; Tel.: +1-404-413-6608

Table S1. Compounds showing no effective inhibition at 500 μM concentrations.

Compound ID	Structure	Δ Florescence	Inhibition %	Gel Images
BW-104	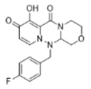	769646	24.75%	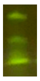
BW-105	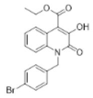	1024479	-0.20%	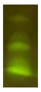
BW-106	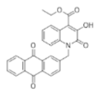	1232554	-20.55%	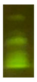
BW-107	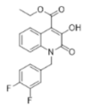	781344	23.58%	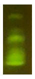
BW-108	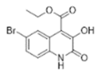	715966	29.97%	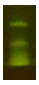
BW-109	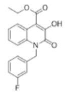	886453	13.3%	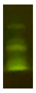
BW-110	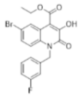	911436	10.86%	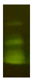
BW-111	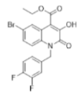	944883	7.59%	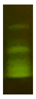
BW-112	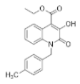	906050	11.39%	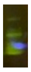

Table S1. Compounds showing no effective inhibition at 500 μM concentrations.

Compound ID	Structure	Δ Florescence	Inhibition %	Gel Images
BW-113	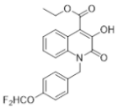	1095742	4.53%	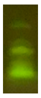
BW-114	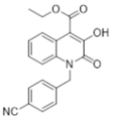	914008	20.36%	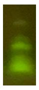
BW-115	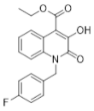	1141144	0.58%	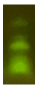
BW-116	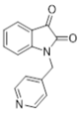	924107	19.49%	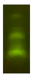
BW-117	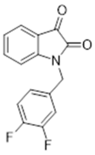	805798	29.79%	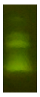
BW-118	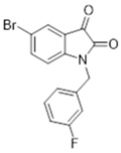	893364	22.16%	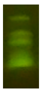
BW-119	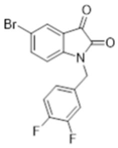	1058398	7.78%	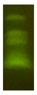
BW-120	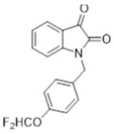	772244	32.72%	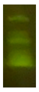
BW-121	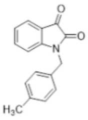	681504	40.62%	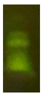
BW-122	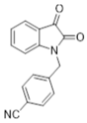	762980	31.26%	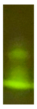

Table S1. Compounds showing no effective inhibition at 500 μM concentrations.

Compound ID	Structure	Δ Florescence	Inhibition %	Gel Images
BW-123	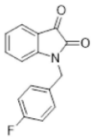	745804	34.85%	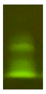
BW-124	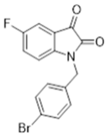	802969	29.85%	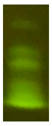
BW-125	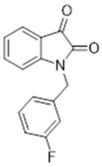	596708	46.24%	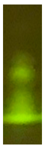
BW-126	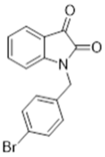	571940	48.47%	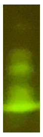
BW-127	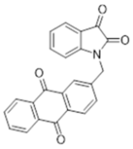	848824	23.53%	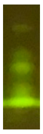
BW-128	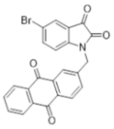	616920	44.42%	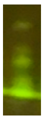
BW-129	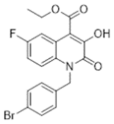	1002343	9.70%	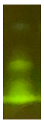
BW-130	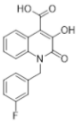	587581	47.06%	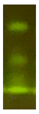
BW-131	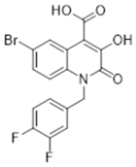	654940	41.00%	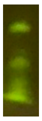

Table S1. Compounds showing no effective inhibition at 500 μM concentrations.

Compound ID	Structure	Δ Florescence	Inhibition %	Gel Images
BW-136	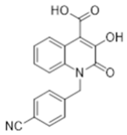	800798	27.85%	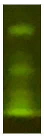
BW-137	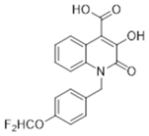	59285	47.81%	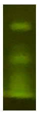
BW-140	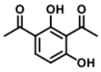	1122239	1.96%	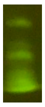
BW-143	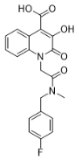	1145676	-0.09%	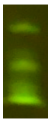
BW-144	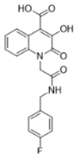	1139264	0.47%	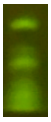
BW-145	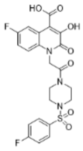	939031	17.97%	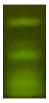
BW-146	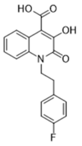	991786	13.36%	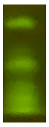
BW-147	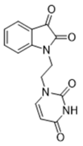	601172	47.48%	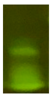

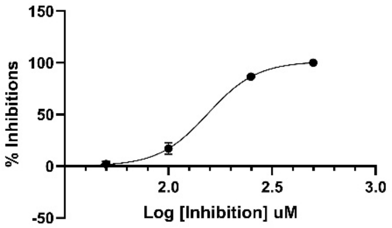

Figure S1: IC50 of BW-149

Docking studies of LASVEN with BW-148 and BXA

By AutoDock 4,2, BW-148 is bound with LASVEN with a predicted binding energy of -7.59 Kcal/mol and Ki of 2.72 μM. The stabilization of the complex was from chelating one of the two Mn^{2+} ions, polar interactions with residues K115, K122 , E102, D89, D66 and the main-chain of V87 and P88. The cation-π stacking is completely abolished in case of LASVEN and possess only non-polar interactions with the thiepane core by residues L48, K44 and S47. The second metal ion does not make any interaction with BW-148, but an additional hydrophobic interaction is brought by the sidechains of H62 and N63 with the cyclopropylmethyl group in the triazinone.

Docking BXA in LASVEN resulted in a pose with predicted binding energy of -5.76 Kcal/mol and Ki of 59.65 μM. BXA showed metal chelation with one of the two Mn^{2+} ions, and polar interactions with residues D89, E102, K115, K122 and the main-chain of C103. No cation-π stacking is observed, yet the triazinone ring oxygen makes a polar interaction with S47, which results in a very different orientation of thiepane core to face the open side of the active side cleft. The thiepane involves only in the hydrophobic interactions with H62, K44, L48 and V87 (Fig 1a and 1b).

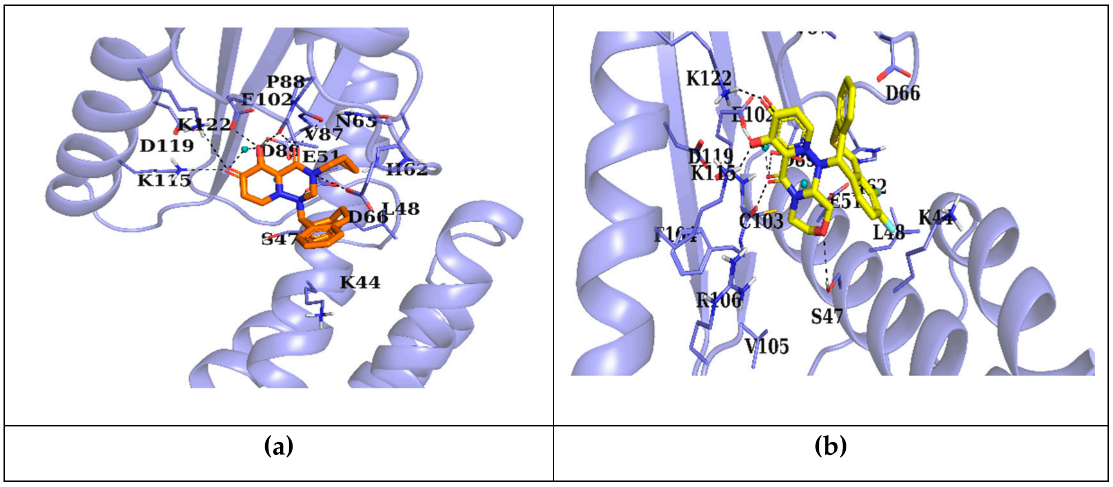

(a)	(b)

Figure S2: Complexes of endonucleases with docked compounds. Active site interactions of LASVEN (light blue) with(a) BW-148 (orange) and(b) BXA (yellow) are shown.

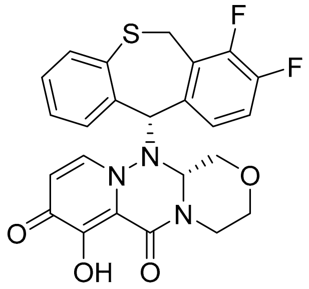

Figure S3: Structure of Baloxavir acid (BXA)
